# Curcumin-Functionalized Gelatin Films: Antioxidant Materials with Modulated Physico-Chemical Properties

**DOI:** 10.3390/polym13111824

**Published:** 2021-05-31

**Authors:** Katia Rubini, Elisa Boanini, Silvia Parmeggiani, Adriana Bigi

**Affiliations:** Department of Chemistry “Giacomo Ciamician”, Alma Mater Studiorum, University of Bologna, Via Selmi 2, 40126 Bologna, Italy; katia.rubini@unibo.it (K.R.); silvia.parmeggiani@unibo.it (S.P.); adriana.bigi@unibo.it (A.B.)

**Keywords:** gelatin, curcumin, swelling, water solubility, DSC, release, antioxidant, radical scavenging activity

## Abstract

In this paper we used curcumin as a functionalizing agent of gelatin films with the aim to get antioxidant materials with modulated physico-chemical properties. To this aim, we prepared gelatin films at different contents of curcumin up to about 1.2 wt%. The as-prepared films, as well as glutaraldehyde crosslinked films, were submitted to several tests: swelling, water solubility, differential scanning calorimetry, X-ray diffraction, mechanical tests and curcumin release. The radical scavenging activity of the as-prepared films is similar to that of free curcumin, indicating remarkable antioxidant properties. All the other tested properties vary as a function of curcumin content and/or the presence of the crosslinking agent. In particular, the films exhibit sustained curcumin release in different solvents. Thanks to its biocompatibility, biodegradability and lack of antigenicity, gelatin uses span from food processing to packaging and biomaterials. It follows that the modulated properties exhibited by the functionalized materials developed in this work can be usefully employed in different application fields.

## 1. Introduction

**Figure 1:**
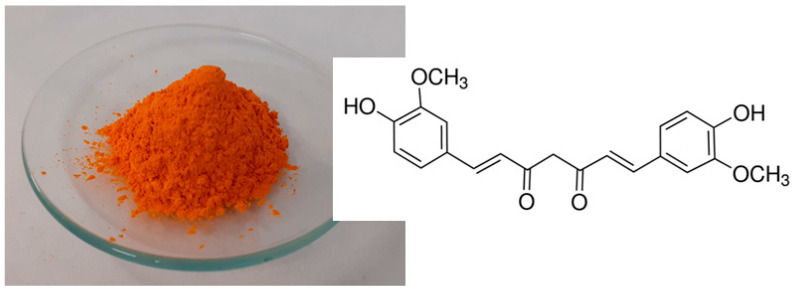
Picture of curcumin orange-colored powder and its molecular structure.

Curcumin—(1E,6E)-1,7-bis(4-hydroxy-3-methoxyphenyl)-1,6-heptadiene-3,5-dione—is a natural polyphenol extracted from the rhizomatous plant *Curcuma Longa* (Turmeric) of the Ginger family [1] (Figure 1). In solution it exhibits a keto-enol tautomerism and shows polymorphism in the solid state, where it exists just as the keto-enol tautomer [2].

Curcumin solutions are generally yellow at pH ≤ 7 and change to red at pH ≥ 8.5 [3]. However, due to its hydrophobicity, it is barely soluble in water. Poor solubility, together with rapid metabolism and systemic elimination, results in poor bioavailability in physiologic conditions [4,5]. On the other hand, curcumin is utilized not only as a spice and coloring agent in food [6], but also in a number of pharmaceutic applications due to its numerous biological activities, including antioxidant, anticancer, anti-inflammatory and antimicrobial properties [4,5,6,7,8]. These desirable characteristics stimulated research aimed to develop polymeric films added with curcumin with the purpose of getting materials with antioxidant and antimicrobial properties [3,9,10,11].

We have previously shown that functionalization of gelatin films with a polyphenol, namely quercetin, provides materials with remarkable antioxidant properties, tailored swelling and mechanical properties [12]. In fact, polyphenols can stabilize proteins through interactions involving hydrogen and covalent bonding, as well as ionic and hydrophobic interactions [13,14,15,16,17]. This is of particular interest in the case of gelatin since this biopolymer, which is abundant, cheap, non-antigenic and biocompatible, is highly soluble in water. It follows that its potential applications, which span from food processing to packaging to biomaterials and pharmaceutics, are severely limited by this drawback. Chemical or physical crosslinking is the strategy usually employed to improve the performances of gelatin materials [18,19,20,21].

Herein, we explored the possibility of imbuing gelatin films with antioxidant properties through functionalization with curcumin. To this aim, we functionalized gelatin films through interaction with water/ethanol solutions at increasing curcumin content. Water solubility, swelling, mechanical and antioxidant properties, as well as curcumin release, were investigated on as-prepared films as well as on films after crosslinking with glutaraldehyde.

## 2. Materials and Methods

### 2.1. Films Preparation

Gelatin from pig skin (280 Bloom, Italgelatine S.p.A.) was used for preparing films with different curcumin contents: 5 g of gelatin were dissolved in 100 mL of water under stirring for 30 min at 40 °C. Films were obtained on the bottom of Petri dishes (diameter = 6 cm) from 7.4 mL of gelatin solution after water evaporation at room temperature. Before complete drying, 7.4 mL of a solution of curcumin (Merck) in water/ethanol (50/50) was placed into each Petri dish that was then sealed for 24 h. Afterwards, curcumin solution was removed, and films were washed with distilled water and completely dried under laminar flow hood. Different amounts of curcumin were used: 0.2, 0.5 and 1 g/L; and obtained films were respectively labeled as G-02, G-05, and G-1. Films without curcumin were prepared as control samples and labeled G-0.

Crosslinked films were prepared following the above procedure until the curcumin solution was removed. After washing each film with water, 7.4 mL of a phosphate buffer solution 0.1 M at pH 7.4 (PBS) containing glutaraldehyde (GTA) 0.15%wt was placed into each Petri dish. After 24 h the solution was removed and films in the dishes were repeatedly washed with glycine solution 1M. Complete drying was attained under laminar flow hood and obtained films were labeled as GTA-02, GTA-05 and GTA-1, as a function of the respective curcumin solution. Crosslinked films without curcumin were prepared as control samples and labeled GTA-0.

### 2.2. Film Characterization

#### 2.2.1. Curcumin Content and Release

For determination of curcumin content, about 100 mg of film was dissolved in 100 mL H_2_O/EtOH 1:1. Absorption spectra were collected with a Varian Cary50Bio instrument (λ = 430 nm). Each analysis was performed in triplicate.

Curcumin release was determined on weighted G or GTA samples (1 cm × 1 cm) placed at the bottom of a vial and added with 5 mL of PBS 0.1M (pH 7.4) or physiological solution (NaCl 0.9%) or H_2_O/EtOH 1:1. Each experiment was performed in triplicate. The release was monitored up to 48 h at room temperature, collecting absorption spectra on supernatant. The spectra were recorded at selected times and the solution was refreshed after each absorbance measurement. Absorption spectra were collected with a Varian Cary50Bio instrument (λ = 430 nm).

#### 2.2.2. Swelling and Water Solubility

For swelling experiments, films were cut into portions of 1 cm × 1cm and were weighted in air-dried conditions. Afterwards they were immersed in PBS 0.1 M pH 7.4 for different periods of time. Wet samples were wiped with filter paper to remove excess liquid and weighed. The amount of absorbed water was calculated as

W (%) = [(W_w_ − W_d_)/W_d_] × 100 (1)

where W_w_ and W_d_ are the weights of the wet and the air-dried samples.

For water solubility determination [22], portions of the films (2 cm × 2 cm) were placed into glass containers with 15 mL of distilled water and subjected to gentle stirring at 65 rpm for 15 h at room temperature. Filtration on filter paper was used to recover the remainder of the undissolved film, which was desiccated at 105 °C for 24 h. The film solubility was calculated as

FS (%) = [(W_0_ − W_f_)/W_0_] × 100 (2)

where W_0_ is the initial weight of the film expressed as dry matter and W_f_ is the weight of the desiccated undissolved rest of the film. Each measurement was performed in triplicate.

#### 2.2.3. Thermal Analysis

Calorimetric measurements were performed using a Perkin Elmer Pyris Diamond differential scanning calorimeter equipped with a model ULSP 90 intra-cooler. Temperature and enthalpy calibration were performed by using high-purity standards (n-decane and indium). The measurements were carried out on dried samples, hermetically sealed in aluminum pans. Heating was carried out at 5 °C min^−1^ in the temperature range from 40 °C to 120 °C. Denaturation temperature was determined as the peak value of the corresponding endothermic phenomena. The value of denaturation enthalpy was calculated with respect to the sample weight.

#### 2.2.4. Mechanical Tests

For mechanical tests, films were immersed into PBS 0.1 M pH 7.4 for 1 min and cut into stripes (3 mm × 30 mm), where thickness was carefully measured for each stripe (values around 0.12 mm) using a Leitz SMLUX- POL microscope.

Stress–strain curves of strip-shaped films were recorded using an INSTRON Testing Machine 4465 with a crosshead speed of 5 mm min^−1^ and the Series IX software package. The Young’s modulus E, the stress at break σ_b_ and the strain at break ε_b_ of the strips were measured.

#### 2.2.5. X-Ray Diffraction

X-ray diffraction analysis was carried out by means of a Panalytical X’Celerator powder diffractometer. Cu Kα radiation was used (40 mA, 40 kV). The 2θ range was from 5° to 40° with a step size of 0.1° and time per step of 200 s.

#### 2.2.6. Radical Scavenging Assay

Antioxidant activity was determined on the basis of curcumin ability to act as radical scavengers toward the 2,2-diphenyl-1-picrylhydrazyl free radical, (DPPH•) (Sigma) [23]. Solutions of GEL-02, GEL-05 and GEL-1 were prepared by dissolving the solid samples in MilliQ water/EtOH and thereafter they were diluted, based on their known curcumin contents, in order to get 10 µM, 30 µM and 50 µM of curcumin concentrations. Dilutions of pure curcumin (Merck), 10 µM, 30 µM and 50 µM, were prepared and used as reference samples. For the assay, 6 mL of each solution (samples and references) were added to 0.2 mL of 1mM DPPH• solution in ethanol, previously clarified by centrifugation (10,000 rpm for 10 min). After an incubation for 30 min at room temperature in darkness, absorbance values (A) were spectrophotometrically measured at 517 nm.

The radical scavenging activity (RSA) was determined through the following equation:

% RSA = (A_0_ − A_x_)/A_0_ × 100 (3)

where A_0_ is the absorbance of the control (containing DPPH• solution without curcumin), and A_x_ is the absorbance in the presence of curcumin (as reference) or of curcumin-containing samples.

#### 2.2.7. Statistical Analysis

Statistical evaluation of data was performed using GraphPad Prism version 5.00 for Windows (GraphPad Software, San Diego, CA, USA). One-way analysis of variance (ANOVA) followed by Dunnett’s Multiple comparison test was used to determine significant differences (*p* < 0.05) among experimental groups and reference samples.

## 3. Results and Discussion

Curcumin solubility in water is very poor, whereas it is soluble in several organic solvents, including ethanol and dimethylsulfoxide [3]. Cooling of gelatin aqueous solutions provokes a partial regain of the triple helix structure characteristic of collagen [24]. The use of DMSO as a solvent hinders this process and prevents gelatin gelification, whereas water/ethanol as a solvent for gelatin yields opaque and non-homogeneous films [12]. Therefore, we utilized a two-step procedure: (i) preparation of gelatin films from an aqueous solution, and (ii) loading of curcumin from a water/ethanol solution.

### 3.1. Curcumin Content

**Figure 2:**
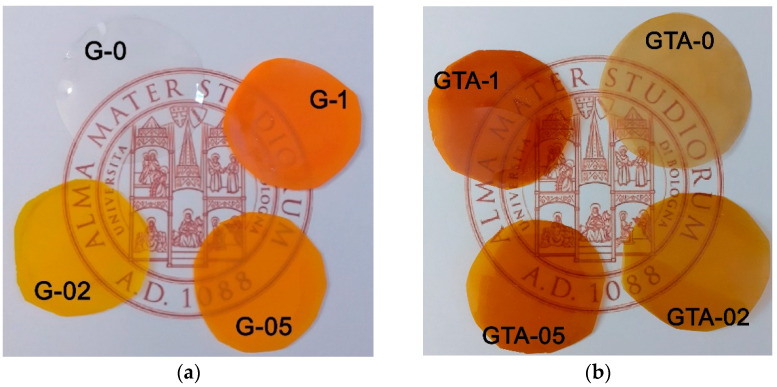
Photographs of films at different curcumin content made from (**a**) gelatin and curcumin; (**b**) gelatin, curcumin and crosslinked with glutaraldehyde solution.

The images in Figure 2a provide a qualitative evaluation of curcumin content of gelatin films submitted to interaction with water/ethanol solutions at different concentrations of the polyphenol. G-0 films are transparent and colorless. Curcumin imbues a yellow color to the films, which becomes more intense when passing from G-02 to G-1.

A further increase of the color intensity occurs after crosslinking with glutaraldehyde (GTA), as shown in Figure 2b. GTA is among the most utilized chemical crosslinking agents for collagenous materials. Although a relatively high content of GTA can be cytotoxic, the low concentration of GTA used in this work does not induce cytotoxicity, whereas it improves mechanical properties and stability of gelatin films [18].

**Figure 3:**
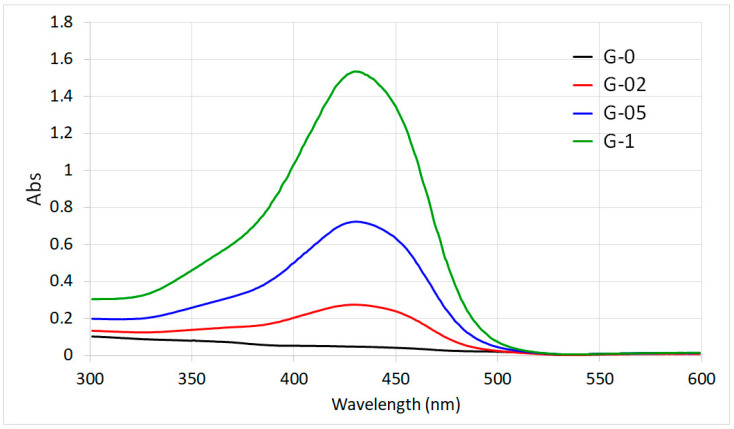
Absorption spectra of film solutions in water/ethanol 1:1.

Curcumin quantitative content was determined through spectrophotometric analysis. Curcumin exhibits an absorption band in the UV region with a maximum at about 265 nm [1] and a further strong absorption band in the visible region, which in water/ethanol solutions falls around 430 nm [25]. The absorption spectra of films dissolved in a water/ethanol mixture reported in Figure 3 clearly show the increase of the intensity of the absorption band in the visible region as a function of curcumin concentration.

Curcumin content determined from evaluation of the absorption spectra (Table 1) increases with its concentration in solution up to about 1.2 wt%. Curcumin content in GTA samples was not measured since the films are not soluble after crosslinking. However, since GTA treatment was performed after curcumin adsorption, it is reasonable to assume the same values as those of G samples.

### 3.2. Swelling and Water Solubility

**Figure 4:**
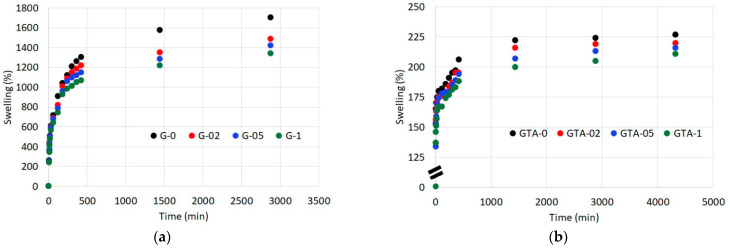
Swelling curves of (**a**) G films and (**b**) GTA films. Each value was determined in triplicate. Standard deviations are comprised of the size of the symbols.

Gelatin undergoes a remarkable swelling in aqueous solution, as shown by the graph reported in Figure 4. Swelling of pristine gelatin films, GEL-0, amounts to about 1700% after 48 h in PBS and further prolongation of immersion time results in complete dissolution of the films. The same trend is observed for G-02, although the presence of curcumin decreases the maximum value of swelling before dissolution down to around 1400%. A further increase of curcumin content not only provokes a further decrease of swelling degree (Figure 4a) but stabilizes the films which maintain their compactness even after a week in PBS. The reduction of swelling in functionalized samples suggests the occurrence of chemical interactions between the functional groups of curcumin and those of gelatin [13,14,15].

A much greater effect on swelling is observed after crosslinking with glutaraldehyde. In the present work, swelling of films crosslinked with GTA reaches a plateau in 24 h, when it assumes remarkably reduced values in comparison with those observed before crosslinking (Figure 4b). The influence of GTA far exceeds that of curcumin so that the values measured for samples at increasing polyphenol content tend to decrease, but are not significantly different.

GTA exerts a great influence also on water solubility: the value of FS (%) of pristine gelatin films decreases down to about one-fifth after crosslinking (Table 1). Moreover, the values of water solubility of crosslinked functionalized samples do not show significant difference as a function of curcumin content, and just the value of GTA-1 is significantly different from that of GTA-0. On the contrary, the influence of the polyphenol is clearly appreciable in non-crosslinked films, which exhibit significantly decreasing FS values when passing from G-0 to G-1. (Table 1).

### 3.3. Differential Scanning Calorimetry

**Figure 5:**
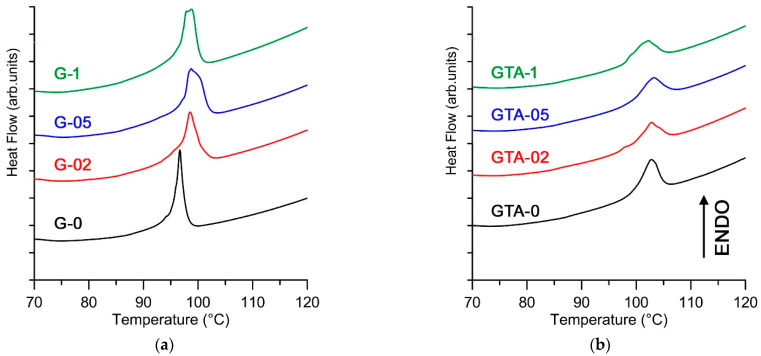
DSC thermograms recorded from (**a**) G films and (**b**) GTA films show the presence of an endothermic peak due to collagen triple helix denaturation.

The DSC plot of G-0 films exhibits an endothermic peak centered at 97 °C (denaturation temperature, T_d_), with a denaturation enthalpy (ΔH_d_) of 23 J/g. The endothermic transition (denaturation) is due to disruption of the portions of triple helix to random coils. In particular, T_d_ and ΔH_d_ are associated with thermal stability and extent of the triple helix structure, respectively [26]. The endothermic peak shows a modest, but appreciable, shift to higher temperatures in functionalized G films, in agreement with an increase of gelatin stability (Figure 5, Table 2), which suggests possible chemical interactions between gelatin and curcumin.

GTA crosslinking provokes a further increase of denaturation temperature and a remarkable reduction of the denaturation enthalpy (Table 2), which can be ascribed to a variation of the relative amounts of hydrogen bonds and covalent crosslinks [26]. As observed for swelling, the effect of GTA crosslinking on denaturation parameters does not allow appreciating any variation due to the presence of curcumin.

### 3.4. Curcumin Release

Curcumin release from the different samples was determined from analysis of the UV-vis absorption spectra. In particular, release was measured in different solvents: water/ethanol, physiological solution and PBS solution.

**Figure 6:**
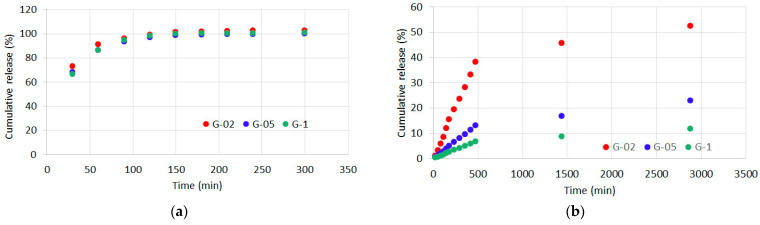

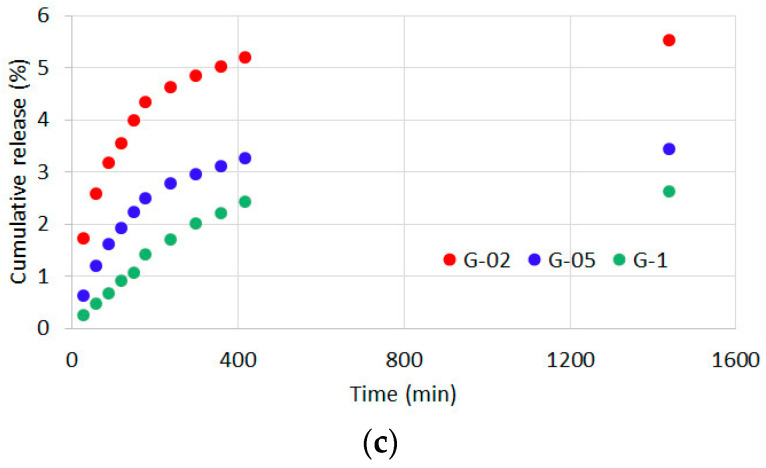
Curcumin cumulative release, expressed as a percentage of the initial content, from G films incubated in (**a**) H_2_O/EtOH solution (1:1); (**b**) physiological solution and (**c**) PBS.

Due to the relatively high solubility of curcumin in H_2_O/EtOH, its release in this solution is fast and almost quantitative. The graph of cumulative release reported in Figure 6a shows that it reaches a plateau in just a couple of hours. The release increases as curcumin initial content increases, and corresponds to complete removal of the polyphenol from the different samples.

At variance, release in physiological solution increases with time up to 0.08 g/g% during the first hours, and reaches values of about 0.10 g/g% in 24 h. In this case, the released amount of curcumin from the different samples does not vary significantly, but it corresponds to different percentages of the polyphenol initial content: up to about 52%, 22% and 12% for G-02, G-05 and G-1, respectively (Figure 6b).

Much smaller values of curcumin release were measured in PBS solution, most likely because the ionic strength of PBS reduces gelatin swelling in comparison to physiological solution [27]. Cumulative release increases with time to reach a steady state in 15–24 h, and indeed it corresponds to small percentages of the initial content of curcumin: about 5.5%, 3.4% and 2.6% for G-02, G-05 and G-1, respectively (Figure 6c).

**Figure 7:**
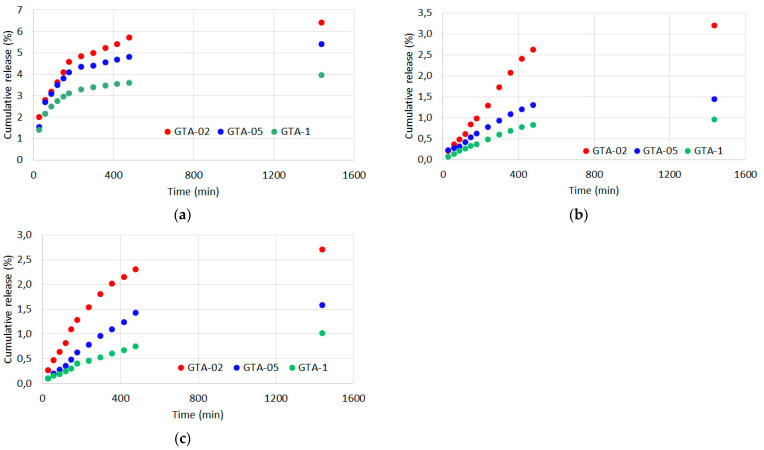
Curcumin cumulative release, expressed as a percentage of the initial content, from GTA films incubated in (**a**) H_2_O/EtOH solution (1:1); (**b**) physiological solution and (**c**) PBS.

As expected, GTA crosslinking dramatically reduces curcumin release. The amount of polyphenol released after 24 h in H_2_O/EtOH is less than 7 wt%, while in physiological solution, as well as in PBS, it just reaches about 3 wt% (Figure 7a–c).

### 3.5. X-Ray Diffraction Analysis

**Figure 8:**
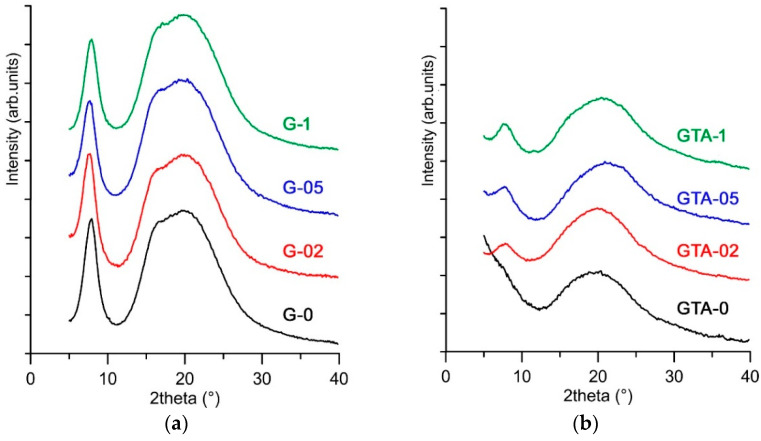
X-rays diffraction patterns of (**a**) G films and (**b**) GTA films.

The X-ray diffraction patterns of un-crosslinked films exhibit two peaks, as shown in Figure 8a: a sharp one at a low angle (about 7.5 °/2θ) and a broad one at about 20 °/2θ, which are due to the characteristic structure of gelatin [28]. The patterns do not show significant modifications as a function of curcumin content.

The low angle peak at about 7.5 °/2θ, which is related to the diameter of the triple helix portions of gelatin, is absent in the XRD pattern of GTA-0 (Figure 8b), suggesting that crosslinking with glutaraldehyde blocks the random coil conformation of gelatin and inhibits the partial regain of triple helix structure occurring during cooling. On the other hand, the relative intensity of this peak increases from GTA-02 to GTA-1, indicating that curcumin counteracts the negative effect of GTA on the partial renaturation of the collagen structure, most likely by reducing the number of available sites for covalent interactions of gelatin with glutaraldehyde.

### 3.6. Mechanical Properties

**Figure 9:**
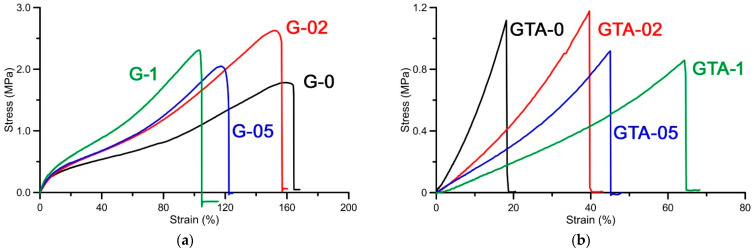
Typical stress–strain curves of (**a**) G films and (**b**) GTA films at different curcumin content.

Functionalization with curcumin influences also the mechanical properties of gelatin films, as shown by the stress-strain curves of gelatin films at increasing polyphenol content (Figure 9a) and by the relative main mechanical parameters reported in Table 3. In particular, the deformation at break, ε_b_, decreases, whereas the values of Young’s modulus, E, increase on increasing curcumin content, suggesting that curcumin interactions with gelatin provokes a stiffening of the films.

The main effect of GTA crosslinking on un-functionalized films is a significant modification of the stress–strain curve (Figure 9b), which involves a dramatic reduction of the value of deformation at break (Table 4). The presence of curcumin in GTA samples interferes with the action of the crosslinking agent in agreement with XRD data, so that the values of ε_b_ show a modest, but significant, increase on increasing polyphenol content, and reduced values of E.

### 3.7. Radical Scavenging Activity

**Figure 10:**
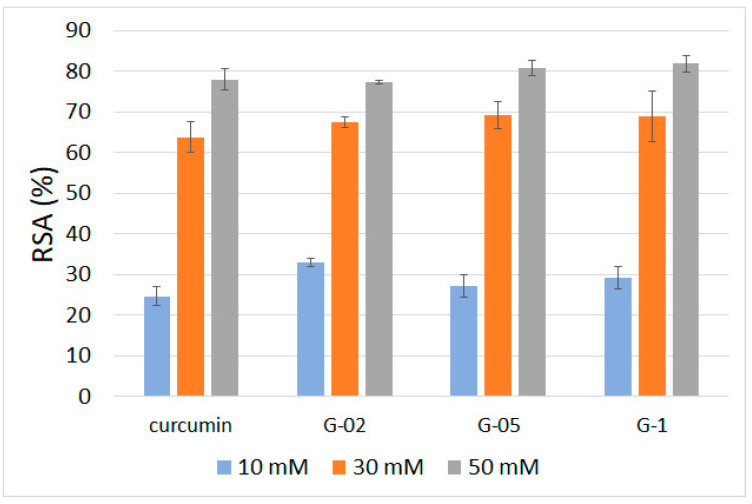
Antiradical activity, expressed as % RSA, of the different samples and pure curcumin toward DPPH•. Bars represent the mean ± SD of two independent measurements.

The ability of curcumin to quench reactive oxygen species is the reason for its antioxidant and anti-inflammatory properties. In this paper, we verify the antioxidant properties of functionalized gelatin films through measurement of their radical scavenging activity (RSA). The test, which is based on 1,1-diphenyl-2-picryl-hydrazyl (DPPH•) assay [29,30] was carried out on different amounts of specimens so as to get different curcumin contents. The results, reported in Figure 10, are compared with those obtained for pure curcumin. Pure curcumin exhibits RSA values increasing from about 25% to about 78% as its concentration increases from 10 to 50 mM.

All the examined samples display RSA values not significantly different from those recorded for pure curcumin, indicating that the antioxidant properties are maintained in functionalized films.

## 4. Conclusions

The amount of curcumin loaded on gelatin films in this work spans from about 0.2 to about 1.2 wt%. Although these might seem modest, they provide the films with modulated properties.

The presence of curcumin reduces swelling and water solubility, causes a modest increase of denaturation enthalpy and reduces the extensibility of the films. Moreover, the films maintain remarkable antioxidant properties as free curcumin and exhibit sustained polyphenol release both in physiological solution and, even more, in PBS.

Crosslinking with glutaraldehyde causes further modulations of the main properties of the films, in particular significant reduction of swelling, water solubility and curcumin release and an increase of thermal stability, as well as variations of mechanical parameters.

In conclusion, the results indicate that gelatin films can be considered good candidates as delivery systems for curcumin, and the wide range of properties shown by the materials developed in this work can be usefully exploited in several applications.

## Figures and Tables

**Table 1:** Curcumin content and water solubility of the functionalized films.

Sample	Curcumin Content (wt%)	FS (%)
G-0		94 ± 5
G-02	0.20 ± 0.02	89 ± 4
G-05	0.55 ± 0.05	86 ± 5
G-1	1.20 ± 0.10	46 ± 3 ^a^
GTA-0		22 ± 1
GTA-02	0.20 ± 0.02	20.3 ± 0.4
GTA-05	0.55 ± 0.05	21.3 ± 0.6
GTA-1	1.20 ± 0.10	19 ± 1 ^a^

^a^ G-1 vs. G-0, G-02, G-05 (*p* < 0.05); GTA-1 vs. GTA-0 (*p* < 0.05).

**Table 2:** Microcalorimetric data: DSC enthalpy variation and temperature of the endothermic transition.

Sample	T_d_ (°C)	ΔH_d_ (J/g)	Sample	T_d_ (°C)	ΔH_d_ (J/g)
G-0	97	23	GTA-0	103	13
G-02	98	23	GTA-02	103	11
G-05	99	23	GTA-05	103	11
G-1	99	24	GTA-1	102	11

**Table 3:** Strain at break, σ_b_, stress at break, ε_b_, and Young’s modulus, E, of gelatin films. Each value is the mean of at least seven determinations and is reported with its standard deviation.

Sample	σ_b_ (MPa)	ε_b_ (%)	E (MPa)
G-0	1.9 ± 0.9	165 ± 35	6.2 ± 0.9
G-02	2.6 ± 0.9	155 ± 10	7.0 ± 1.9
G-05	2.2 ± 1.2	125 ± 9 ^a,b^	7.6 ± 1.1
G-1	2.3 ± 0.5	105 ± 8 ^a^	8.9 ± 0.8 ^a^

ε_b_: ^a^ G-1 vs. G-0, G-02, G-05; G-05 vs. G-02 (*p* < 0.001). ^b^ G-05 vs. G-0 (*p* < 0.05). E: ^a^ G-1 vs. G-0 (*p* < 0.001).

**Table 4:** Strain at break, σ_b_, stress at break, ε_b_, and Young’s modulus, E, of GTA gelatin films. Each value is the mean of at least seven determinations and is reported with its standard deviation.

Sample	σ_b_ (MPa)	ε_b_ (%)	E (MPa)
GTA-0	1.1 ± 0.5	18 ± 5	6.5 ± 0.9
GTA-02	1.2 ± 0.5	40 ± 10 ^a^	2.1 ± 0.8 ^a^
GTA-05	0.9 ± 0.6	45 ± 12 ^a^	1.7 ± 0.3 ^a^
GTA-1	0.9 ± 0.5	64 ± 18 ^a,b^	1.1 ± 0.8 ^a^

ε_b_: ^a^ GTA-1, GTA-05, GTA-02 vs. GTA-0 (*p* < 0.001); ^b^ GTA-1 vs. GTA-02 (*p* < 0.05). E: ^a^ GTA-1, GTA-05, GTA-02 vs. GTA-0 (*p* < 0.001).

## Data Availability

The data presented in this study are available on request from the corresponding author.
